# Modeling and Optimizing the Synthesis of Urea-formaldehyde Fertilizers and Analyses of Factors Affecting these Processes

**DOI:** 10.1038/s41598-018-22698-8

**Published:** 2018-03-14

**Authors:** Yanle Guo, Min Zhang, Zhiguang Liu, Xiaofei Tian, Shugang Zhang, Chenhao Zhao, Hao Lu

**Affiliations:** 1 National Engineering Laboratory for Efficient Utilization of Soil and Fertilizer Resources, College of Resources and Environment, Shandong Agricultural University, Taian, Shandong 271018 China; 2 State Key Laboratory of Nutrition Resources Integrated Utilization, Kingenta Ecological Engineering Group Co., Ltd., Linshu, 276700 China

## Abstract

Previous research into the synthesis of urea-formaldehyde fertilizers was mostly based on orthogonal experimental designs or single factor tests; this led to low precision for synthesis and relatively large ranges of parameters for these processes. To obtain mathematical response models for the synthesis of urea-formaldehyde fertilizers with different nitrogen release properties, a central composite design (CCD) of response surface methodology was used in our research to examine the effects of different reaction times, temperatures, and molar ratios on nitrogen insoluble in either hot or cold water. Our results showed that nitrogen insoluble in cold or hot water from urea-formaldehyde fertilizers were mainly affected by urea: formaldehyde molar ratios. Also, quadratic polynomial mathematical models were established for urea-formaldehyde. According to the models, the optimal process parameters which maximize cold-water-insoluble nitrogen and minimize hot-water-insoluble nitrogen for the synthesis of urea formaldehyde were as follows urea: formaldehyde molar ratio was 1.33, reaction temperature was 43.5 °C, and reaction time was 1.64 h. Under these conditions, the content of cold-water-insoluble nitrogen was 22.14%, and hot-water-insoluble nitrogen was 9.87%. The model could be an effective tool for predicting properties of urea-formaldehyde slow release fertilizers if the experimental conditions were held within the design limits.

## Introduction

In recent decades, the development of controlled or slow release fertilizers (SRFs) has been fast and became a focus of fertilizers research^1^. Urea-formaldehyde (UF) has become one of the most commonly used slow-release fertilizers worldwide^2^. Urea-formaldehyde fertilizers offer good physical properties and slow release rates; they can promote the formation of an aggregated soil structure, improve soil permeability, and increase penetrating power into crop roots. With fast, long-term functioning, the nitrogen use efficiency may exceed 50%^3–5^. In the soil, this may result from microbial hydrolysis into ammonium, carbon dioxide, and water, which involves plant absorption and use. The result would be complete degradation of the fertilizer, which would be environmentally friendly, and thus confer a unique advantage to using these slow-release fertilizers^6,7^.

Urea and formaldehyde was used to form polymers under certain conditions of temperature, molar ratio, and reaction time. The release period was affected by the degree of polymerization^8^. There has been previous domestic and foreign research and development into urea-formaldehyde slow release fertilizers that more concentrated in single factor and orthogonal design, the deep-level research of forecasting mathematical models is not enough^9–11^. The research into urea-formaldehyde production lacked a systematic and comprehensive approach; hence, it is urgent to advance this technology. Research into the synthesis of urea-formaldehyde mainly focused on a single-factor, while the range of process parameters was larger.

The single factor test can only be optimized for one variable at a time, the orthogonal design can only obtain the relative optimal solutions of different variables and limited light combinations, the two-order polynomial model between the parameters and the response value of the whole variable with all combinations cannot be obtained, so the final optimization result may not be the best scheme for urea-formaldehyde synthesis. Many studies showed that suitable conditions for synthesizing urea-formaldehyde fertilizers involved a urea formaldehyde molar ratio of 1.2–1.5, reaction temperatures of 30–50 °C, and reaction times of 1–2 h, but the interactions between reaction factors were not clear^5,12^. Simultaneously, there was an urgent need to solve the problem of varying the synthesis of urea-formaldehyde fertilizers so they would have dissolution rates that were tailored to the varying needs of different crops. Another problem involves how to reduce the proportion of quick-acting components while increasing the proportion of slow-acting components^13^. This was directly related to the insolubility of nitrogen^14^ from the fertilizer in hot or cold water.

The response surface method was used to model data from experiments using the “reasonable design method”. The response surface method involved building a model of a surface using continuous variables. It has been used to analyze optimal conditions in systems with multi-factor interaction analyses including among different factors. It was widely used in different scientific fields to solve problems with a multivariate experimental design and methods of statistical analyses and was effective in helping to solve these problems^15,16^. To the best of our knowledge, however, no previous study has used response surface methodology to determine optimal methods for synthesis of urea-formaldehyde fertilizers. In the present study, we used the method of central composite design to develop and analyze a method for synthesis of urea-formaldehyde fertilizers. The mathematical model was established using response surface methodology, and then its validity was verified. Parameters for optimal synthesis of the fertilizer were determined using the response surface method. The results provide a theoretical basis for rapidly extracting nitrogen, which has been insoluble in either hot or cold water, from the urea-formaldehyde products.

## Results and Discussions

The experimental design and results for experimental trials were determined (Table 1). ANOVAs helped to produce the quadratic models for response surfaces, which elucidated the fitness, accuracy, and significance of the models in addition to the effects of interaction results and individual variables on the responses^17^ (Table 2).

**Table 1:** The experimental design and responses based on experimental trials.

Trial	Independent variables			Responses (dependent variables)^a^	
	*X*_1_	*X*_2_(°C)	*X*_3_ (h)	*Y*_1_ (%)	*Y*_2_ (%)
1	1.20	30.00	2.00	26.69	21.19
2	1.20	50.00	1.00	23.83	17.90
3	1.35	40.00	1.50	21.33	8.65
4	1.35	40.00	1.50	21.78	8.20
5	1.35	40.00	1.50	21.78	9.33
6	1.35	56.82	1.50	21.34	10.76
7	1.35	40.00	1.50	21.75	8.97
8	1.35	40.00	1.50	21.57	10.46
9	1.50	30.00	2.00	13.30	6.02
10	1.20	50.00	2.00	25.88	17.70
11	1.60	40.00	1.50	13.61	3.42
12	1.35	40.00	1.50	20.54	8.73
13	1.50	50.00	1.00	19.19	7.79
14	1.35	23.18	1.50	23.31	15.10
15	1.35	40.00	0.66	20.02	9.01
16	1.10	40.00	1.50	29.07	22.80
17	1.20	30.00	1.00	26.46	16.75
18	1.50	30.00	1.00	16.40	6.71
19	1.35	40.00	2.34	21.09	8.68
20	1.50	50.00	2.00	18.41	7.40

^a^Nitrogen insoluble in cold water (*Y*_1_) or in hot water (*Y*_2_).

**Table 2:** ANOVAs for the regression models.

Source^a^		Sum of squares	Mean square	F value	Df.	Prob > F	Significance^b^
Model	*Y*_1_	303.78	33.75	51.54	9	<0.0001	**
	*Y*_2_	527.79	58.64	38.71	9	<0.0001	**
*X*_1_	*Y*_1_	277.49	277.49	423.76	1	<0.0001	**
	*Y*_2_	447.93	447.93	295.71	1	<0.0001	**
*X*_2_	*Y*_1_	0.096	0.096	0.15	1	0.7094	
	*Y*_2_	3.77	3.77	2.49	1	0.1456	
*X*_3_	*Y*_1_	2.915E-003	2.915E-003	4.451E-003	1	0.9481	
	*Y*_2_	0.50	0.50	0.33	1	0.5795	
*X*_1_ *X*_2_	*Y*_1_	16.07	16.07	24.55	1	0.0006	*
	*Y*_2_	2.88	2.88	1.90	1	0.1980	
*X*_1_ *X*_3_	*Y*_1_	4.74	4.74	7.24	1	0.0226	*
	*Y*_2_	3.54	3.54	2.34	1	0.1574	
*X*_2_ *X*_3_	*Y*_1_	2.14	2.14	3.27	1	0.1006	
	*Y*_2_	2.35	2.35	1.55	1	0.2409	
*X*_1_^2^	*Y*_1_	0.071	0.071	0.11	1	0.7493	
	*Y*_2_	37.94	37.94	25.05	1	0.0005	*
*X*_2_^2^	*Y*_1_	1.12	1.12	1.70	1	0.2210	
	*Y*_2_	35.02	35.02	23.12	1	0.0007	*
*X*_3_^2^	*Y*_1_	1.74	1.74	2.66	1	0.1341	
	*Y*_2_	0.19	0.19	0.13	1	0.7308	
Residual	*Y*_1_	6.55	0.65		10		
	*Y*_2_	15.15	1.51		10		
Lack of fit	*Y*_1_	5.38	1.08	4.62	5	0.0591	
	*Y*_2_	12.09	2.42	3.95	5	0.0788	
Pure error	*Y*_1_	1.16	0.23		5		
	*Y*_2_	3.06	0.61		5		
Corr. total	*Y*_1_	310.33			19		
	*Y*_2_	542.94			19		

^a^*Y*_1_: R^2^ = 0.9789, Adj R^2^ = 0.9599; *Y*_2_: R^2^ = 0.9721, Adj R^2^ = 0.9470.

^b^*significant, **highly significant.

### Effects of urea-formaldehyde reaction time, temperature, and molar ratio on the levels of cold-water-insoluble nitrogen

The effects of reaction time, temperature, and urea-formaldehyde molar ratios on the design array for variable *Y*_1_ for nitrogen insoluble in cold water were evaluated through regression analyses (Table 1). The resulting quadratic equation relating reaction time, temperature, and urea/formaldehyde molar ratios were determined (Equation 1).

1 $$\begin{array}{rcl}{Y}_{1} & = & 93.75437-44.04921{X}_{1}-1.64519{X}_{2}+13.91988{X}_{3}\\  &  & +\,0.94500{X}_{1}{X}_{2}-10.26667{X}_{1}{X}_{3}+0.10350{X}_{2}{X}_{3}-3.11181{{X}_{1}}^{2}\\  &  & +\,(2.78234{\rm{E}}-003){{X}_{2}}^{2}-1.39022{{X}_{3}}^{2}.\end{array}$$

**Figure 1:**
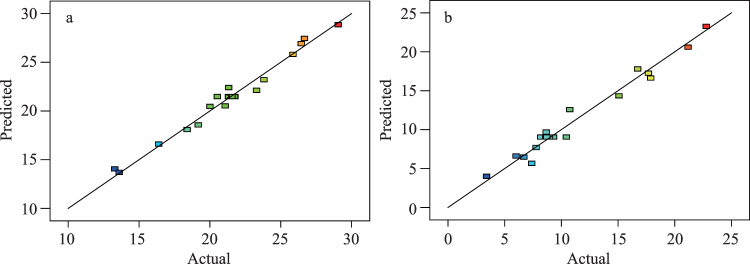
Relationships between actual and predicted responses for nitrogen insoluble in cold water *Y*_1_, (**a**) or in hot water *Y*_2_, (**b**).

Here *Y*_1_ was the percentage of nitrogen insoluble in cold water, and *X*_1_(urea/formaldehyde molar ratio), *X*_2_(reaction temperature), and *X*_3_(reaction time) were the independent variables. The correlation coefficients R^2^ and adjusted R^2^ were evaluated with the latter reflecting an adjustment for number of model parameters relative to the number of points in the study. R^2^ for *Y*_1_ was 97.89% indicated a strong relationship between experimental and predicted values (Fig. 1a). However, the adjusted R^2^ for *Y*_1_ (95.99%) also reflects the influence of independent variables.

**Figure 2:**
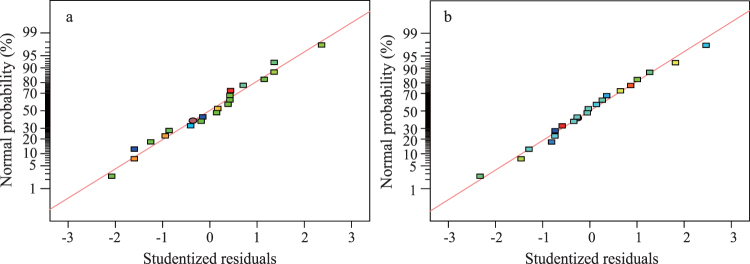
Normality plots for residuals from analyses of nitrogen insoluble in cold water *Y*_1_, (**a**) or in hot water *Y*_2_, (**b**).

A useful diagnostic method in regression analysis is a plot of normal probability for the residual: an approximately linear pattern represents normality in the error term, whereas a non-linear pattern shows non-normality^18^. For nitrogen insoluble in cold water, a highly normal linear pattern was found; hence, none of the responses strongly deviated from normality^19^ (Fig. 2a).

Based on ANOVAs for the regression models, results were highly significant for the percentage of nitrogen insoluble in cold water (*Y*_1_, Table 2). Here, the computed F-value (51.54) was greater than F_0.05_,_9,8_ (3.39) implying the model was significant at *p* ≤ 0.05, or a level greater than 95%. In addition, values for the lack of fit were used to estimate data variation around each response model. Each lack-of-fit F-value compared values for changes in pure error with those for changes in the lack-of-fit; an insignificant result indicated the model fit the data well^12^. For the percentage of nitrogen insoluble in cold water (*Y*_1_), the lack-of-fit results were insignificant with an F-value (4.62) lower than F_0.05,3,5_ (5.41) and a *p*-value of 0.0591; again, the model fit the data very well.

The significance of individual effects for interaction, quadratic, and linear sources or models were also tested (Table 2). Generally, results that were significant for each term of the mathematical model to the response variable indicated a strong effect and that it was an important term for the model^19^. Statistical analyses including ANOVAs (Table 2) suggested that the most highly significant term was the urea-formaldehyde molar ratio, which had two terms with significant interactions (*X*_1_*X*_2_ and *X*_1_*X*_3_).

### Effects of urea-formaldehyde reaction time, temperature, and molar ratio on the levels of hot-water-insoluble nitrogen

For the interactive effects of reaction time, temperature, and molar ratio (independent variables) on the dependent variable, hot-water-insoluble nitrogen, a quadratic regression equation was determined (Equation 2).

2 $$\begin{array}{rcl}{Y}_{2} & = & 216.64307-235.59277{X}_{1}-1.67699{X}_{2}+15.31510{X}_{3}\\  &  & +\,0.40000{X}_{1}{X}_{2}-8.86667{X}_{1}{X}_{3}-0.10850{X}_{2}{X}_{3}+72.11578{{X}_{1}}^{2}\\  &  & +\,0.015590{{X}_{2}}^{2}+0.45880X{{X}_{3}}^{2}.\end{array}$$

Correlation coefficients R^2^ and adjusted-R^2^ were used to test the fit of the model (Equation 2). R^2^ was 0.9721 indicating the model predicted the response well because R^2^ was close to 1^20,21^. The value of the adjusted-R^2^ (0.9470) was also very high indicated that a satisfactory adjustment of the mathematical model to the test data indicating a very significant response model^22^. The R^2^ and adjusted-R^2^ for the models were each close to 1 indicating a close match between estimated and experimental values (Fig. 1b). The plot residuals also tended to cluster around the diagonal line for the predicted result indicated the normality assumptions were satisfactory (Fig. 2b). Additional analyses by ANOVAs showed the F-value for the response model for *Y*_2_ (38.71) was highly significant (*p* < 0.0001; Table 2). The F_0.05,3,5_ for lack-of-fit was insignificant because the resulting value (5.41) was greater than the predicted F-value (3.95) (Table 2). The lack-of-fit *p*-value (0.0788) indicated there was 7.88% chance that a lack-of-fit F-value would occur because of randomness or “noise”. This is greater than the generally accepted level by the agricultural sciences (5%), hence, the lack-of-fit was not significant compared with the pure error value^12,23^.

**Table 3:** Specifications for the factors used in experimental analyses.

Variables	Codes	Units	Levels		
			−1	0	1
Molar ratio (urea/ formaldehyde)	*X*_1_	1	1.2	1.35	1.5
Reaction temperature	*X*_2_	°C	30	40	50
Reaction time	*X*_3_	h	1	1.5	2

Changes in responses for *X*_1_(urea/formaldehyde molar ratio), *X*_2_(reaction temperature), and *X*_3_(reaction time) were shown by negative and positive coefficients for the primary effects (Equation 2). The absolute value of the coefficients (Equation 2) supported a strong correlation with their effects size^24,25^. Central composite design of response surface methodology (CCD/RSM) experiments revealed a sequence for significance as follows: molar ratios > reaction times > reaction temperatures. Results of ANOVAs and other statistical analyses showed that the molar ratio for urea-formaldehyde was a significant linear term, and *X*_1_^2^, *X*_2_^2^ were two significant quadratic terms, which showed positive interactions (Table 2). Here, the most highly significant term was the urea-formaldehyde molar ratio.

## Discussion

**Figure 3:**
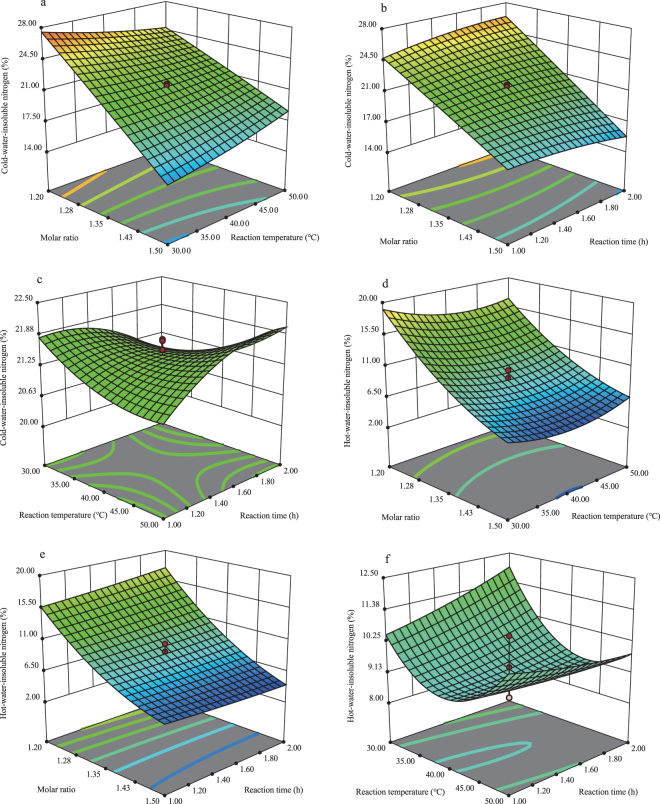
Response surfaces showing the effects of each parameter on levels of cold-water-insoluble nitrogen or hot-water-insoluble nitrogen.

**Figure 4:**
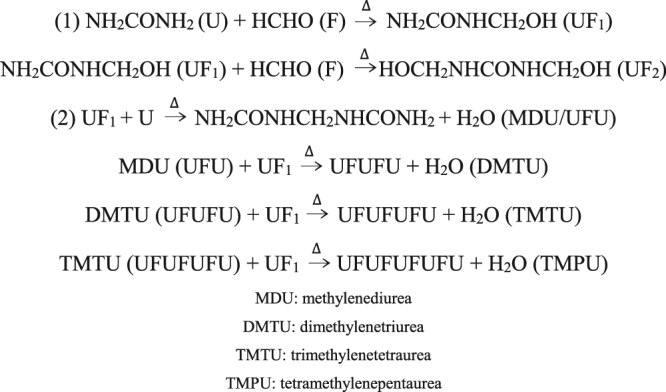
The reaction of urea (U) with formaldehyde (F).

The three-dimensional response surfaces were based on equation (1) and helped to understand the interactive and main effects of independent variables (Fig. 3a–c). The surface showed interactive effects of urea-formaldehyde molar ratios and reaction temperatures and suggested that the levels of cold-water-insoluble nitrogen decreased with increasing molar ratios of urea-formaldehyde^26^ (Fig. 3a). The reaction of formaldehyde and urea in aqueous solutions initially form monomethylolurea, which may react with another formaldehyde molecule resulting in dimethylolurea. The reaction of urea with formaldehyde is reversible in a strongly alkaline solution. But under acidic conditions, methyleneureas including methylenediurea, dimethylenetriurea, and trimethylenetetraurea are formed by polymerizing reactions. Hence, non-reacted urea, monomethylolurea, dimethylolurea, methylenediurea, and dimethylenetriurea were the molecules containing cold-water-soluble nitrogen. When the molar ratio was 1.5, the concentration of formaldehyde was relatively small and decreased the formation of monomethylolurea, which in turn supplied the raw materials for the second step of the reaction. Hence, the polymethylene urea showed a lower rate of polymerization resulting in a lower concentration of cold-water-insoluble nitrogen at a molar ratio of 1.5 than at 1.2 (Fig. 4)^27,28^.

Considering reaction temperatures, the cold-water-insoluble nitrogen increased with increasing temperatures when the urea-formaldehyde molar ratio was 1.50, but the maximum value of the cold-water-insoluble nitrogen decreased with the increased of the reaction temperature. The reaction of formaldehyde and urea in aqueous solution was a two-step process (Fig. 4). Under acidic conditions, it was exothermic, and not conducive to polymerization as the temperature increased.

There was a significant interaction between molar ratio and reaction time for cold-water-insoluble nitrogen (Fig. 3b). Molar ratio was the most important factor affecting levels of cold-water-insoluble nitrogen, whereas reaction time had almost no effect. Cold-water-insoluble nitrogen levels increased markedly with decreasing urea/formaldehyde molar ratios.

Based on levels of cold-water-insoluble nitrogen, the interaction of temperature and time was not significant. This is suggested by a relatively gentle slope on the response surface (Fig. 3c).

Molar ratios have often significantly affected the cold-water-insoluble nitrogen of resulting urea-formaldehyde products; hence the results of the present study were similar to previous research^1,13^. In the present study, changes in the reaction temperature or time did not significantly affect the levels of cold-water-insoluble nitrogen resulting from urea-formaldehyde^29^.

Using 3D graphs to represent interactions and other effects has been highly recommended to allow interpretation of models and experimental results^30,31^. The present study successfully used 3D plots obtained from mathematical models to explain the results (Fig. 3d–f); hence we support these suggestions from other authors.

Based on significant interaction between reaction temperature and molar ratio, for a given reaction temperature (*X*_2_), the levels of hot-water-insoluble nitrogen increased with decreasing molar ratio (Fig. 3d). Considering reaction temperatures, however, the hot-water-insoluble nitrogen decreased initially then increased with increasing temperatures (Fig. 3d). The first step of the reaction between urea and formaldehyde was an endothermic reaction, followed by an exothermic reaction for the second step; the hot-water-insoluble nitrogen generated in the second step, the first step is the foundation of the second step, and higher temperature is in favor of the first step of the reaction, lowering the temperature is conducive to the second reaction, the complexity of this reaction process may help account for there is a relatively optimal temperature makes which maximize the nitrogen insoluble in cold water and minimize the nitrogen insoluble in hot water^21,22^; further research could help in understanding these processes.

Considering interactive effects between molar ratio and reaction time for hot-water-insoluble nitrogen, molar ratio was the most variable factor and it decreased with increasing levels of hot-water-insoluble nitrogen (Fig. 3e). However, reaction time had almost no effect on the levels of hot-water-insoluble nitrogen. In addition, the response surface slope for the reaction of hot-water-insoluble nitrogen (*Y*_2_) to temperature and time showed the interaction of *X*2 and X3 was not significant (Fig. 3f).

### Verification of the Model

Optimal conditions for the synthesis of urea-formaldehyde fertilizer were predicted using Design Expert software for numerical optimization. Here, the objective was to maximize the yield of nitrogen insoluble in cold water and minimize the yield of nitrogen insoluble in hot water by choosing optimal values from the response surface^16^. The resulting optimal conditions were as follows: urea-formaldehyde molar ratio 1.33, temperature 43.5 °C, and time 1.64 h. Predicted values for nitrogen insoluble in cold and in hot water were 22.14% and 9.87%, respectively. Given practical considerations, the optimal conditions were adjusted as follows^32^: molar ratio 1.33, temperature 44 °C, time 1.6 h, and nitrogen insoluble in cold and in hot water were 21.36% and 9.27%, respectively. Specific conditions for organic synthesis reactions were chosen in part to facilitate testing the validity of analytical methods using response surfaces. Because of good correlation within the results, the mathematical models determining these responses were helpful in finding expected optimal values^33^. These models may be effective for predicting levels of nitrogen insoluble in hot or cold water if experimental conditions were held within limits provided by the model assumptions^19^.

## Conclusion

Quadratic polynomial equations were developed using response surfaces based on central composite design (CCD) techniques to predict optimal conditions for the synthesis of urea-formaldehyde fertilizer. Analyses of variance (ANOVAs) indicated the models for nitrogen insoluble in cold or hot water were each significant at the 95% confidence level (*p* ≤ 0.05), which is widely accepted as significant by different scientific disciplines. Reaction time, temperature, and urea-formaldehyde molar ratio all strongly affected the levels of nitrogen insoluble in cold or hot water; here, molar ratio was the most highly significant variable.

Central composite design of response surface methodology (CCD/RSM) experiments led to the determination of the following hierarchy for levels of significance: molar ratio > reaction time > reaction temperature. Based on the response surfaces, optimal conditions for maximizing nitrogen insoluble in cold water and minimizing the nitrogen insoluble in hot water were as follows: molar ratio 1.33, reaction temperature 43.5 °C, and reaction time 1.64 h. Under these conditions, levels of nitrogen insoluble in cold water and in hot water reached 22.14% and 9.87%, respectively. Main benefits from optimizing the synthesis of urea-formaldehyde fertilizers included a better understanding of the reaction processes and more effective production of the fertilizer using different options. These can allow for a maximum sustained release of nitrogen or a more convenient, faster release as needed.

## Experimental Section

### Chemicals and other materials

All solutions used were of analytical quality or higher; they included urea, formaldehyde (37%), hydrochloric acid, sodium hydroxide, ammonium chloride, and others (Taian Putian Chemical Co., Taian, Shandong, China).

### Instruments and measurements

Equipment used included an AIM600 Digestion Block (AIM 600 Digestion Block System; AIM Lap Automatic Technologies, Clontarf, Australia); motorized stirrer (#JJ-1, Shanghai Shuangjie Co., Shanghai, China); ultrapure water system (#D24UV, Millipore Co., Billerica, MA, USA); automatic kjeldahl instrument (#UDK159, VELP Co., Italy); analytical balance (Secura #224–1CN, Sartorius Group, Germany); analytical balance (Quintix #2102–1CN, Sartorius Group, Germany); thermostatically controlled water bath (#DK-8D, Shanghai, China); and a laboratory reactor system (#LR-2.ST, IKA Co., Germany).

### Synthesis procedures

**Figure 5:**
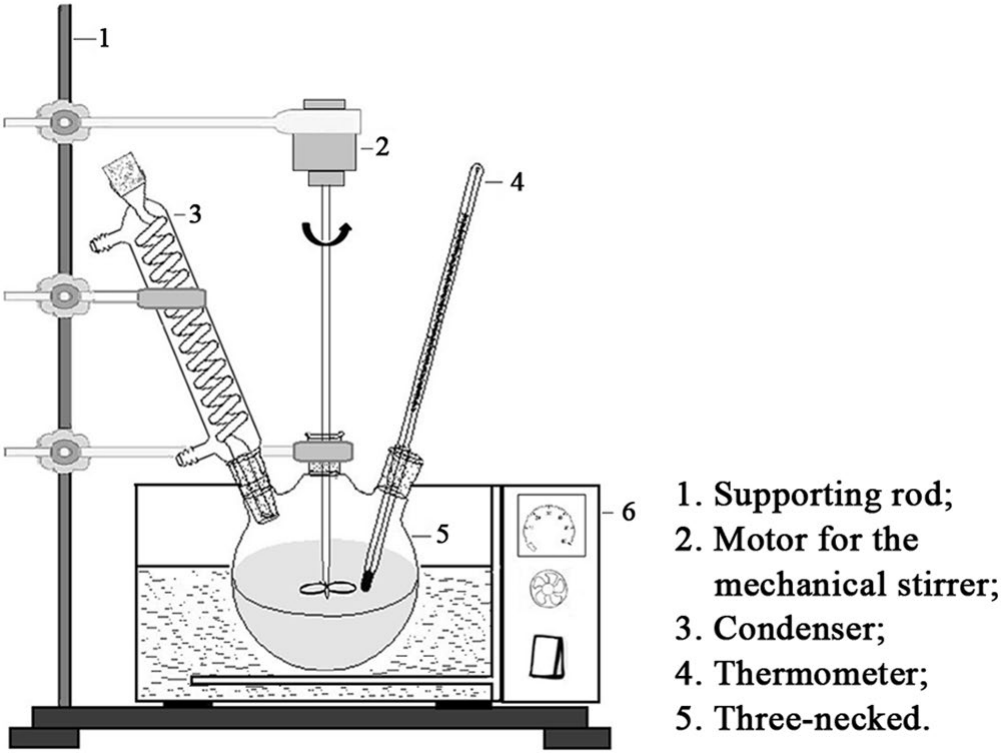
Experimental setup.

Reactions for polymerization of urea and formaldehyde solution were performed in 250-mL, three-necked, round-bottom flasks. For each test, urea and formaldehyde solutions were added to a three-necked flask, which was equipped with a funnel, reflux condenser, thermometer, and motorized stirrer (Fig. 5). The agitation rate was a constant 200 RPM for all experiments^34^. After the urea was dissolved, the pH of the system was adjusted to 9.0 by adding 1 mol/L of NaOH solution. Then, the flask with solution was heated in the water bath to a certain temperature and duration of heating, which were pre-set based on an experimental design table. The design table also helped to determine the amount of ammonium chloride subsequently added into the flask; the pH of the solution was simultaneously adjusted to 5.0 by adding HCl solution. After maintaining these conditions for 1 h, all contents of the flask were transferred into a large glass dish and dried at 90 °C in a drying oven. Finally, the urea-formaldehyde was synthetized and prepared for the analyses.

### Analytical methods

Nitrogen insoluble in cold water was extracted using a phosphate buffer solution (pH 7.5) for 15 min at 25 °C, and the hot-water-insoluble nitrogen was extracted using an identical solution for 30 min at 100 °C. All the resulting samples were crushed to allow passage through an 18-mesh Tyler standard sieve and were analyzed according to a standard procedure of the Chinese government (Chemical Industry Standard #HG/T 4137–2010, urea aldehyde slow release fertilizers, People’s Republic of China).

### Experimental design and data analyses

Based on previous studies, we did not use the one-factor-at-a-time technique for analyses. To determine the optimal reaction conditions, a five-level, three-factor central composite design (CCD) was used (Table 3), and 20 experimental runs were performed^32^. Variables used to represent synthesis of the urea-formaldehyde fertilizer included urea/formaldehyde molar ratio (1.2–1.5), reaction time (1–2 h), and temperature (30–50 °C). The results were analyzed using response surface methodology and a second-order polynomial equation (Equation 3).

3 $$Y=\alpha 0+\sum _{i=1}^{n}\alpha iXi+\sum _{i=1}^{n}\alpha iiX{i}^{2}+\sum _{i=1}^{n}\sum _{j=i+1}^{n}\alpha ijXiXj$$

Here, the variables included the response of nitrogen insoluble in either hot or cold water (*Y*); *X*_i_ and *X*_j_, the independent variables; *α*_0_ an offset term; *α*_i_, for linear effects; *α*_ij_, the first-order interaction effect; and *α*_ii_, the squared effect.

The design of experiments, building of regression models, and data analyses were performed with Design Expert software (Version 11.0.3.0, Stat-Ease Inc., Minneapolis, MN, USA). To determine the ideal conditions for synthesis of urea-formaldehyde fertilizer, analyses of variance (ANOVAs) were used followed by regression analyses and plotting the response surfaces. In addition, factorial analyses helped to test for two-factor interactions using two-way ANOVAs, followed by one-way ANOVAs using mean separation techniques after determining whether interaction or no-interaction occurred. Three-dimensional surface charts showing responses of two independent variables were produced from the data using response surface methodology. Other ANOVAs or regression analyses led to linear or quadratic equations and their variables. In all analyses, *p* < 0.05 was considered significant. When optimal conditions for reactions were predicted, the tests were repeated three times to check for consistency^35^.

## Electronic supplementary material

Supporting Information
