# Supplementary material for: Modeling and Optimizing the Synthesis of Urea-formaldehyde Fertilizers and Analyses of Factors Affecting these Processes
Source: Sci Rep. 2018 Mar 14;8:4504. doi: 10.1038/s41598-018-22698-8 (PMC5852125; doi:10.1038/s41598-018-22698-8)
Supplement: Supplementary file 1 — Supporting Information [file 41598_2018_22698_MOESM1_ESM.pdf]

Supporting Information

Title: Modeling and Optimizing the Synthesis of Urea-formaldehyde Fertilizers and Analyses of Factors Affecting these Processes

Authors: Yanle Guo[1], Min Zhang[1, 2*], Zhiguang Liu[1*], Xiaofei Tian[1], Shugang Zhang[1], Chenhao Zhao[1], Hao Lu[1]

[1]National Engineering Laboratory for Efficient Utilization of Soil and Fertilizer Resources, National Engineering and Technology Research Center for Slow and Controlled Release Fertilizers, College of Resources and Environment, Shandong Agricultural University, Taian, Shandong, 271018, China.

[2]State Key Laboratory of Nutrition Resources Integrated Utilization, Kingenta Ecological Engineering Group Co., Ltd. Linshu 276700, China.

Key words: urea-formaldehyde, response surface methodology, mathematical model, optimization

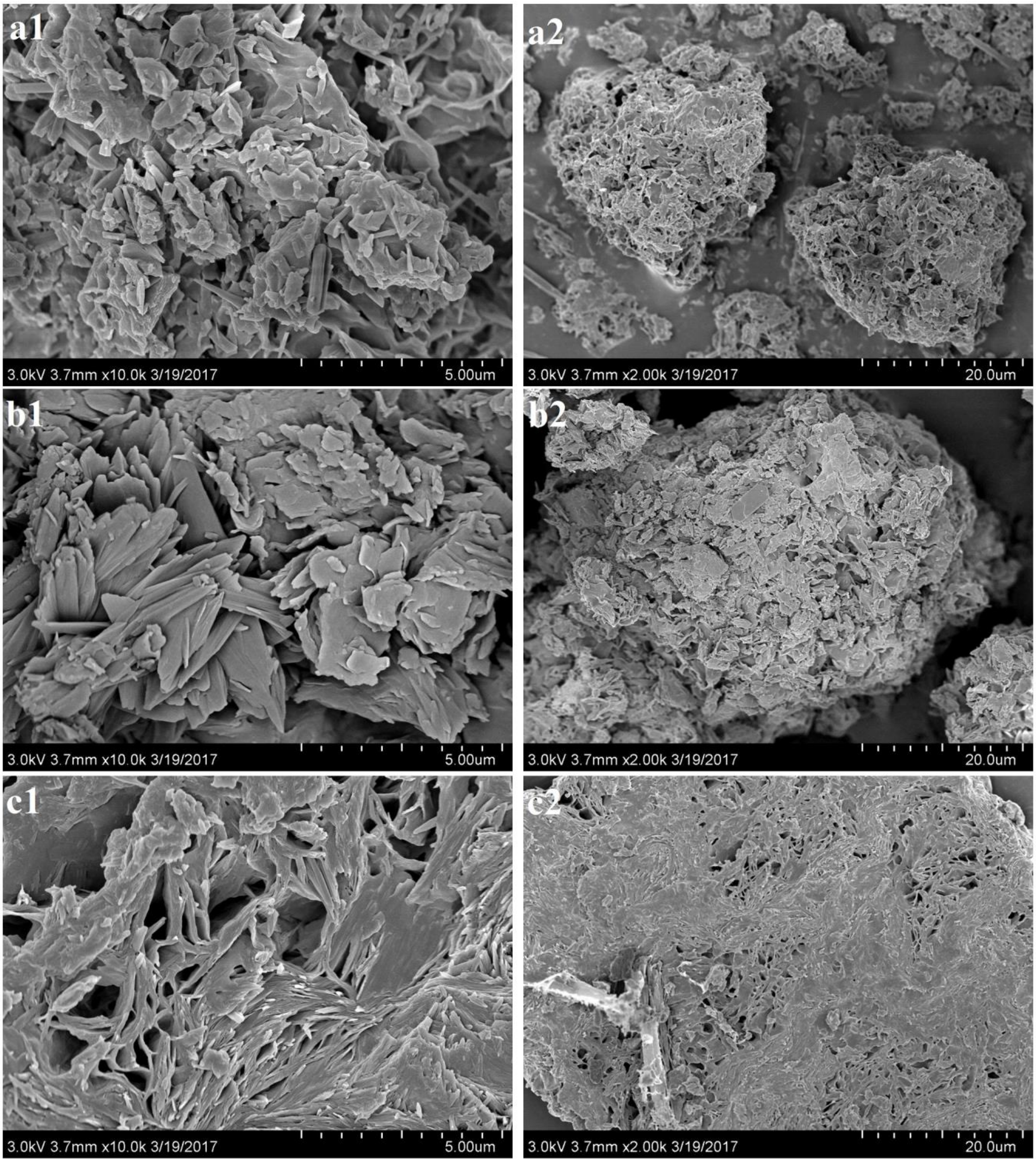

Fig.S1 Scanning electron microscope images of urea-formaldehyde fertilizer (a. Sample of Trial 1; b. Sample of Trial 8; c. Sample of Trial 11)
